# Risk factors and clinical impact of sodium and potassium disorders in community-acquired pneumonia

**DOI:** 10.1186/s12890-026-04322-y

**Published:** 2026-04-29

**Authors:** Güzide Tomas, Şeyma Başlılar, Ayşe Çapar

**Affiliations:** 1 Chest Disease Clinic, İstanbul Sultan Abdülhamid Han Training and Research Hospital, Istanbul, Türkiye; 2 Anesthesiology and Intensive Care Medicine, İstanbul Sultan Abdülhamid Han Training and Research Hospital, Istanbul, Türkiye

**Keywords:** Community-acquired pneumonia, Electrolyte imbalance, Hyponatremia, Hypokalemia, Mortality

## Abstract

**Introduction and aim:**

Dysnatremia and dyskalemia are common electrolyte disturbances in patients hospitalized with community-acquired pneumonia (CAP) and have been associated with increased disease severity and adverse clinical outcomes. This study aimed to evaluate the relationship between sodium and potassium disturbances at hospital admission and clinical outcomes, including morbidity and early and late mortality, in patients with CAP.

**Materials and methods:**

A total of 998 patients hospitalized with CAP were retrospectively analyzed. Demographic characteristics, comorbidities, admission laboratory parameters, CURB-65 scores, intensive care unit (ICU) admission, ventilatory support requirements, in-hospital mortality, and six-month mortality were recorded. Patients were classified according to the presence and type of electrolyte disturbances at hospital admission and compared accordingly.

**Results:**

The mean age of the study population was 71.4 ± 15.6 years, and 46.0% (*n* = 459) were female. Mean serum sodium and potassium levels at admission were 138.9 ± 5.6 mmol/L and 4.38 ± 0.72 mmol/L, respectively. Hyponatremia was the most frequent electrolyte abnormality (22.7%). Patients with potassium disturbances had significantly higher procalcitonin levels (*p* < 0.001). The need for ICU and MV and in-hospital mortality was higher in patients with hypernatremia and hypokalemia, while 6 months mortality was more frequent in patients with hyperkalemia(*p* < 0,001) and hospital stay was longer among patients with hypernatremia (*p* = 0.002).

**Conclusion:**

Electrolyte disturbances at hospital admission are common in patients with CAP and are associated with greater disease severity and increased supportive care requirements. These abnormalities should be interpreted primarily as markers of underlying physiological stress and comorbidity burden rather than independent predictors of mortality. Early recognition of dysnatremia and dyskalemia may support clinical risk awareness and prompt evaluation of potentially reversible contributing factors during inpatient management of CAP.

**Supplementary Information:**

The online version contains supplementary material available at 10.1186/s12890-026-04322-y.

## Introduction and aim

Community-acquired pneumonia (CAP) remains one of the leading causes of morbidity and mortality worldwide, particularly among elderly patients and those with multiple comorbidities [1]. In hospitalized patients, systemic disturbances may develop due to the infection itself, underlying organ dysfunction, or treatment-related factors. Among these, abnormalities in serum sodium and potassium levels including hypo or hypernatremia and hypo or hyperkalemia are common. Electrolyte disturbances in CAP may result from reduced oral intake, volume depletion, diuretic use, renal impairment, or dysregulated antidiuretic hormone secretion, such as in the syndrome of inappropriate antidiuretic hormone secretion (SIADH) [2]. studies suggest that sodium and potassium abnormalities may be associated with adverse outcomes. Ravioli et al. reported hyponatremia and hypokalemia rates of 28.8% and 15.6%, respectively, in hospitalized CAP patients, with hyponatremia linked to prolonged hospital stay [3]. Similarly, Zhao et al. demonstrated that deviations in serum electrolyte levels, including both sodium and potassium abnormalities, were associated with worse short-term outcomes in patients hospitalized with acute illness [4].

In critically ill populations, electrolyte disturbances are of particular clinical relevance, as they may contribute to arrhythmias, delayed liberation from mechanical ventilation, and multiorgan dysfunction [5]. Despite this, available data regarding the prevalence of sodium and potassium disorders in hospitalized CAP patients, the clinical factors associated with their occurrence, and their relationship with short- and medium-term outcomes remain limited and sometimes inconsistent.

Therefore, the present study aimed to evaluate the prevalence of sodium and potassium disturbances at hospital admission in patients hospitalized with community-acquired pneumonia, to identify clinical and laboratory factors associated with these abnormalities, and to examine their associations with key clinical outcomes, ICU admission, need for mechanical ventilation, length of hospital stay, in-hospital mortality, and six-month mortality.

## Materials and methods

This retrospective, cross-sectional study was conducted in the pulmonology clinic and respiratory intensive care unit of Sultan Abdülhamid Han Training and Research Hospital and included patients hospitalized with CAP between January 1, 2017, and June 1, 2024. The study was approved by the Ethics Committee of Süreyyapaşa Chest Diseases and Thoracic Surgery Training and Research Hospital (approval number: 2024–8, dated June 13, 2024). As the study was retrospective in nature and conducted using anonymized patient data, the requirement for informed consent was waived by the ethics committee.

Community-acquired pneumonia (CAP) was diagnosed based on clinical, radiological, and laboratory findings in accordance with the American Thoracic Society/Infectious Diseases Society of America (ATS/IDSA) guidelines [6]. Patients diagnosed with hospital-acquired pneumonia, COVID-19 pneumonia, or other viral pneumonias were excluded. Viral pneumonias were excluded to ensure a more homogeneous study population, as viral respiratory infections may involve distinct pathophysiological mechanisms affecting electrolyte balance, including differences in inflammatory response, antidiuretic hormone regulation, and fluid management [7, 8].

Patients with active malignancy were excluded from the study. Active malignancy was defined as the presence of ongoing cancer-related treatment, including chemotherapy, radiotherapy, immunotherapy, or targeted therapy at the time of hospitalization. Patients with a history of malignancy, including previous lung cancer, who were not receiving active oncological treatment at the time of admission were included in the study.

**Fig. 1:**
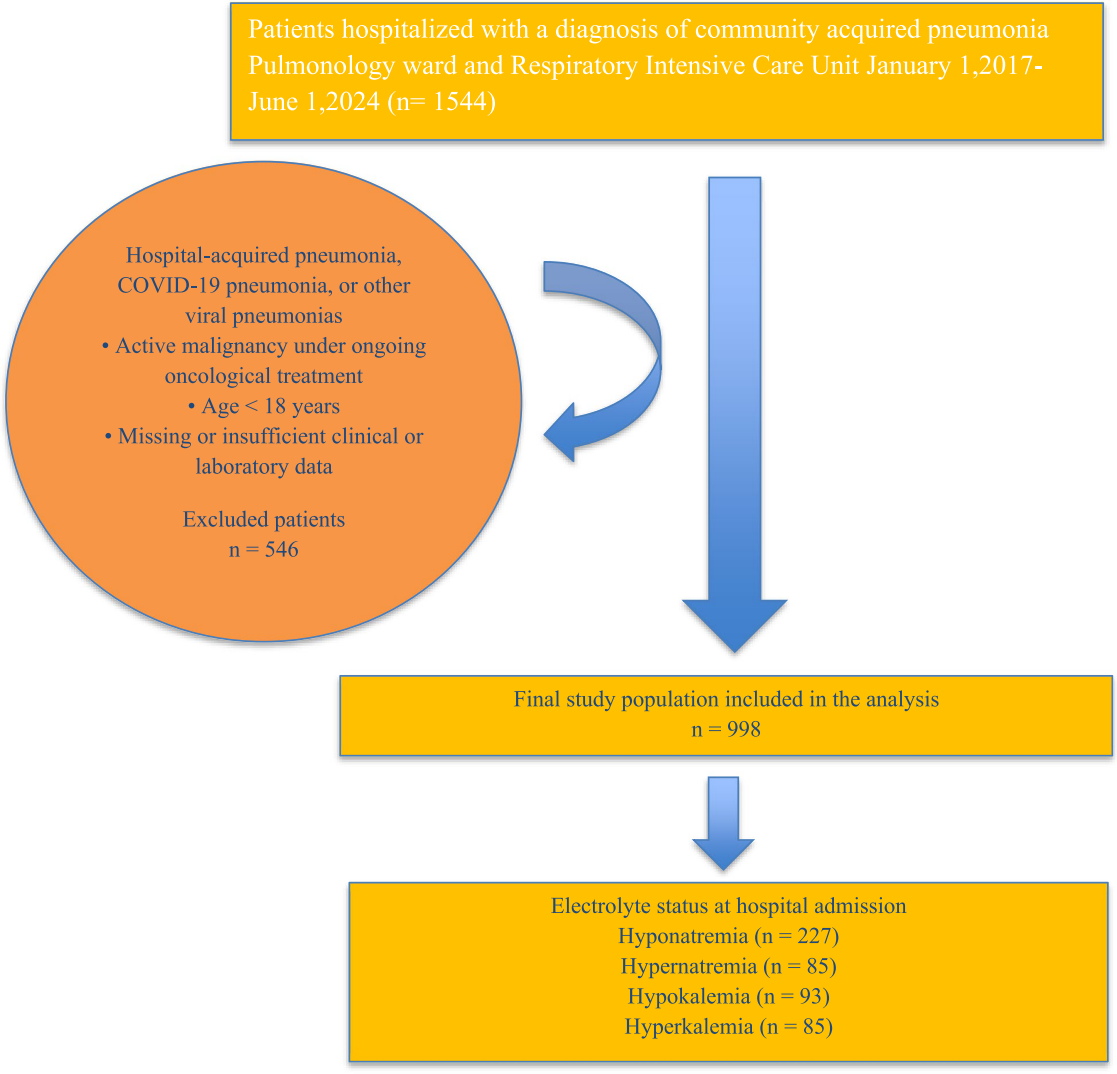
Flow chart of patient selection and inclusion in the study

Additional exclusion criteria included insufficient or missing clinical or laboratory data. Patients under 18 years of age were also excluded (Fig. 1).

Laboratory measurements; Blood samples were collected at hospital admission and analyzed in the central laboratory of the study center using standardized automated analyzers in accordance with the manufacturer’s instructions. Serum sodium, potassium, glucose, creatinine, albumin, C-reactive protein, and procalcitonin levels were measured as part of routine clinical care. In patients who underwent arterial blood gas analysis, serum osmolality values were recorded when available.

The sodium and potassium values analyzed in this study represent the first laboratory measurements obtained at hospital admission. Serum sodium levels were not corrected for glucose concentration. Serum osmolality was assessed in patients with hyponatremia when available and used to classify hyponatremia as hypoosmolar, normoosmolar, or hyperosmolar.

Microbiological evaluation; Sputum and blood cultures were obtained at the discretion of the treating physician in accordance with routine clinical practice. Sputum samples were processed using standard microbiological techniques and cultured on appropriate solid media, including blood agar and MacConkey agar. Blood cultures were collected using standard aseptic techniques and incubated in automated blood culture systems.

Isolated microorganisms were identified using conventional microbiological methods in the hospital microbiology laboratory. Due to the limited number of positive cultures, microbiological findings were analyzed descriptively.

Patients were excluded if they met one or more of the predefined exclusion criteria.

Electrolyte abnormalities were analyzed as non mutually exclusive categories, and the reported counts may overlap within individual patients.

Demographic and clinical data, including age, sex, comorbidities (such as chronic heart failure, chronic kidney disease, and diabetes mellitus), medication use (including diuretics and angiotensin-converting enzyme inhibitors), smoking status, nursing home residence, and functional status were recorded. Previous pneumonia was defined as any documented episode of pneumonia requiring hospitalization at any time prior to the index admission. Laboratory parameters obtained at hospital admission included serum sodium and potassium levels, complete blood count, C-reactive protein (CRP), procalcitonin, serum creatinine, estimated glomerular filtration rate (eGFR), and albumin levels. The sodium and potassium levels analyzed in this study were the first laboratory values measured at the time of hospital admission. Length of hospital stay, ICU admission, need for invasive or non-invasive mechanical ventilation, in-hospital mortality, and six-month mortality were recorded.

Electrolyte disturbances were defined based on admission laboratory values. Hyponatremia was defined as a serum sodium level < 135 mmol/L and hypernatremia as > 145 mmol/L. Hypokalemia was defined as a serum potassium level < 3.5 mmol/L and hyperkalemia as > 5.0 mmol/L. Patients were initially grouped according to the presence or absence of any electrolyte disturbance and subsequently categorized into four subgroups: hyponatremia, hypernatremia, hypokalemia, and hyperkalemia. For patients with available data, serum osmolality was evaluated in those with hyponatremia and classified as hypoosmolar, normoosmolar, or hyperosmolar hyponatremia.

### Statistical analysis

Statistical analyses were performed using IBM SPSS Statistics for MacOS, version 30.0 (IBM Corp., Armonk, NY, USA). Categorical variables were presented as numbers and percentages [n (%)], while continuous variables were expressed as mean ± standard deviation for normally distributed data and as median with interquartile range [median (IQR)] for non-normally distributed data. For comparisons among groups, one-way ANOVA was used when the assumption of homogeneity of variances was met, and Welch ANOVA was applied when this assumption was violated. The Kruskal–Wallis test was used for continuous variables that did not follow a normal distribution. When significant differences were observed, appropriate post hoc tests were performed based on distribution and variance characteristics (Bonferroni for ANOVA, Games–Howell for Welch ANOVA, and Dunn–Bonferroni for Kruskal–Wallis). Categorical variables were compared using the chi-square test. To identify independent predictors associated with electrolyte disturbances, multivariable logistic regression analyses were performed. Variables considered clinically relevant and those showing statistically significant differences in univariate analyses were entered into the multivariable models using the enter method. Prior to model construction, multicollinearity among independent variables was assessed using variance inflation factor (VIF) and tolerance statistics. In all models, the maximum VIF values were below 2.5 and the minimum tolerance values were above 0.40, indicating no evidence of significant multicollinearity (Supplementary Table 1). Results were reported as odds ratios (ORs) with 95% confidence intervals (CIs). A *p*-value of < 0.05 was considered statistically significant for all analyses.

## Results

A total of 998 hospitalized patients with community-acquired pneumonia (CAP) were included in the final analysis. The mean age of the study population was 71.4 ± 15.6 years, and 46.0% (*n* = 459) were female. The most frequent comorbidities were chronic lung disease (49.5%), diabetes mellitus (27.9%), and chronic heart failure (21.7%). The mean serum sodium and potassium levels at hospital admission were 138.9 ± 5.6 mmol/L and 4.38 ± 0.72 mmol/L, respectively. Hyponatremia was the most common electrolyte abnormality (22.7%), followed by hypokalemia (9.3%), hypernatremia (8.5%), and hyperkalemia (8.5%).

The median length of hospital stay was 6 days (IQR 4–9). Overall, 14.4% of patients required intensive care unit (ICU) admission, 9.3% required non-invasive ventilation, and 6.8% required invasive mechanical ventilation. In-hospital mortality was 6.2%, and six-month mortality was 6.3%.

### Sodium disturbances

Clinical characteristics and outcomes; Patients were categorized into three groups according to serum sodium levels at admission: hyponatremia (*n* = 227), normonatremia (*n* = 686), and hypernatremia (*n* = 85). Comparisons of demographic characteristics, comorbidities, and clinical outcomes are presented in Table 1. None of the patients had severe hyponatremia (defined as serum sodium level < 120 mmol/L).

**Table 1:** Distribution of demographic characteristics, comorbidities, and clinical outcomes according to serum sodium levels

Variables (*N* = 998)	Hyponatremia (*n* = 227)	Normonatremia(*n* = 686)	Hypernatremia (*n* = 85)	*p*-value	Post-hoc
Age, years	72 ± 15	71 ± 16	77 ± 14	0.001	2–3
ACE inhibitor use, n (%)	52 (22.9)	98 (14.3)	25 (29.4)	< 0.001	
Diuretic use, n (%)	65 (28.6)	85 (12.4)	28 (32.9)	< 0.001	
Sex, n (%)				0.013	
Female	103 (45.4)	304 (44.3)	52 (61.2)		
Male	124 (54.6)	382 (55.7)	33 (38.8)		
Chroniclung disease,n (%)	115 (50.7)	343 (50.0)	36 (42.4)	0.381	
Diabetes mellitus, n (%)	72 (31.7)	194 (28.3)	12 (14.1)	0.008	
Chronic heart failure, n (%)	61 (27.0)	134 (19.5)	22 (25.9)	0.039	
Chronic kidney disease, n (%)	21 (9.3)	59 (8.6)	6 (7.1)	0.828	
Historyof malignancy,n (%)	16 (7.0)	32 (4.7)	7 (8.2)	0.203	
Immunosuppression, n (%)	20 (8.8)	42 (6.1)	8 (9.4)	0.258	
Aspiration pneumonia, n (%)	19 (8.4)	59 (8.6)	15 (17.6)	0.022	
Nursing home residence, n (%)	5 (2.2)	11 (1.6)	3 (3.5)	0.440	
Functional status, n (%)	31 (13.7)	104 (15.2)	24 (28.2)	0.005	
CURB-65 score, n (%)				< 0.001	
0–1	89 (39.2)	275 (40.1)	17 (20.0)		
2	79 (34.8)	275 (40.1)	37 (43.5)		
≥ 3	59 (26.0)	136 (19.8)	31 (36.5)		
History of pneumonia, n (%)	65 (28.6)	174 (25.4)	22 (25.9)	0.623	
Macrolide use, n (%)	111 (48.9)	328 (47.8)	39 (45.9)	0.891	
Days of macrolide therapy	6.2 ± 3.2	6.3 ± 2.9	6.6 ± 3.5	0.732	
Days of empirical antibiotic therapy	6 (4–8)	6 (4–8)	6 (4–10)	0.417	
Carbapenem resistance, n (%)	5 (6.1)	23 (9.2)	7 (17.9)	0.111	
ICU admission, n (%)	28 (12.3)	87 (12.7)	29 (34.1)	< 0.001	
Need for invasive mechanical ventilation, n (%)	11 (4.8)	38 (5.5)	19 (22.4)	< 0.001	
Need for non-invasive ventilation, n (%)	22 (9.7)	62 (9.0)	9 (10.6)	0.877	
Length of hospital stay, days	6 (4–10)	6 (4–9)	7 (5–11)	0.013	2–3
In-hospital mortality, n (%)	11 (4.8)	32 (4.7)	19 (22.4)	< 0.001	
6-month mortality, n (%)	11 (4.8)	42 (6.1)	10 (11.8)	0.077	

Continuous variables are presented as mean ± standard deviation or median (interquartile range), as appropriate. Comparisons among the three groups were performed using one-way ANOVA with Bonferroni correction, Welch ANOVA with Games–Howell post-hoc test, or Kruskal–Wallis test with Dunn–Bonferroni correction, depending on data distribution and variance homogeneity. Categorical variables were compared using the chi-square test; Fisher’s exact test or Monte Carlo exact test was applied when expected cell counts were insufficient

Immunosuppression was defined as ongoing systemic immunosuppressive or immunomodulatory therapy at the time of hospital admission, including systemic corticosteroids and other immunosuppressive agents prescribed for chronic inflammatory or autoimmune diseases. Altered functional status was defined based on clinical documentation indicating reduced functional capacity or dependence in activities of daily living at the time of hospital admission. No formal functional scoring system was applied

There was a significant difference in age among the three groups (*p* = 0.001); post-hoc analysis demonstrated that patients with hypernatremia were significantly older than those with normal sodium levels. Diuretic use differed significantly across groups (*p* < 0.001) and was most frequent among patients with hyponatremia. The prevalence of diabetes mellitus was higher in the hyponatremia group (31.7%) compared with the normonatremia and hypernatremia groups (*p* = 0.008). Chronic heart failure was also more frequent among patients with hyponatremia (*p* = 0.039).

Regarding clinical outcomes, significant differences were observed in ICU admission (*p* < 0.001), invasive mechanical ventilation requirement (*p* < 0.001), length of hospital stay (*p* = 0.013), and in-hospital mortality (*p* < 0.001). Post-hoc analyses indicated that these adverse outcomes were predominantly observed in the hypernatremia group. Six-month mortality was numerically higher in patients with hypernatremia but did not reach statistical significance (*p* = 0.077).

Laboratory findings; Admission laboratory parameters according to sodium categories are shown in Table 2. Estimated glomerular filtration rate (eGFR) differed significantly among groups (*p* = 0.015), with post-hoc analysis demonstrating lower eGFR values in the hypernatremia group compared with both hyponatremia and normonatremia groups. Serum albumin levels also differed significantly (*p *= 0.019), with higher values observed in patients with hypernatremia compared with normonatremic patients.

**Table 2:** Distribution of admission laboratory parameters according to serum sodium levels

Variables (*N* = 998)	Hyponatremia^1^ (*n* = 227)	Normonatremia^2^ (*n* = 686)	Hypernatremia^3^ (*n* = 85)	*p*-value	Post-hoc*
eGFR, mL/min/1.73 m^2^	68.2 ± 30.5	68.3 ± 30.8	58.3 ± 24.7	0.015	1–3; 2–3
Serum creatinine, mg/dL	1.0 (0.8–1.2)	1.0 (0.8–1.3)	1.0 (0.9–1.35)	0.468	
Serum albumin, g/L	29 (26–34)	29 (25–34)	32 (27–36)	0.019	2–3
Serum osmolality, mOsm/kg	266 (259–275.2)	–	–	–	
Serum potassium, mmol/L	4.4 (4.0–4.9)	4.4 (3.9–4.8)	4.2 (3.5–4.6)	0.001	1–3; 2–3
C-reactive protein (CRP), mg/L	101.9 ± 71.5	101.8 ± 80.8	101.4 ± 75.6	0.999	
Procalcitonin, ng/mL	0.2 (0–0.7)	0.2 (0–1.1)	0.1 (0.1–0.9)	0.792	
Arterial pH	7.4 (7.4–7.5)	7.4 (7.4–7.5)	7.4 (7.3–7.5)	0.462	
Serum lactate, mmol/L	1.5 (1.0–2.2)	1.6 (1.2–2.3)	1.7 (1.2–2.6)	0.152	

Continuous variables are presented as mean ± standard deviation or median (interquartile range), as appropriate. Comparisons among the three groups were performed using one-way ANOVA with Bonferroni correction, Welch ANOVA with Games–Howell post-hoc test, or Kruskal–Wallis test with Dunn–Bonferroni correction, depending on data distribution and variance homogeneity

*Abbreviations*: *eGFR* estimated glomerular filtration rate, *CRP* C-reactive protein.

### Osmolality subgroups among patients with hyponatremia

Among patients with hyponatremia, serum osmolality data were available in a subset of cases. Hyponatremia was classified as hypoosmolar in 88 patients, normoosmolar in 24 patients, and hyperosmolar in 9 patients (Table 3). No significant differences were observed among osmolality subgroups with respect to age, sex, diuretic use, or the prevalence of chronic heart failure (all *p* > 0.05).

**Table 3:** Distribution of demographic and clinical characteristics according to osmolality subgroups among patients with hyponatremia

Variables	Hypoosmolar Hyponatremia (*n* = 88)	Normoosmolar Hyponatremia (*n* = 24)	Hyperosmolar Hyponatremia (*n* = 9)	*p*-value
Age, years	71 ± 17	74 ± 9	74 ± 14	0.917
Sex, n (%)				0.398
Female	44 (50.0)	9 (37.5)	3 (33.3)	
Male	44 (50.0)	15 (62.5)	6 (66.7)	
ACE inhibitor use, n (%)	23 (26.1)	7 (29.2)	2 (22.2)	0.915
Diuretic use, n (%)	24 (27.3)	10 (41.7)	2 (22.2)	0.344
Chronic heart failure, n (%)	25 (28.4)	7 (29.2)	2 (22.2)	0.918

Continuous variables are presented as mean ± standard deviation. Categorical variables are presented as number and percentage [n (%)]. Comparisons among osmolality subgroups were performed using appropriate parametric or non-parametric tests based on data distribution.

*Abbreviations ACE* Angiotensin-converting enzyme, *CHF* Chronic heart failure.

Serum potassium levels varied significantly across sodium categories (*p* = 0.001), with lower potassium levels observed in the hypernatremia group. No significant differences were detected among groups with respect to C-reactive protein, procalcitonin, arterial pH, or lactate levels (all *p *> 0.05).

### Potassium disturbances

Clinical characteristics and outcomes; Patients were classified into three groups according to serum potassium levels at hospital admission: hypokalemia (*n* = 93), normokalemia (*n* = 796), and hyperkalemia (*n* = 109). Demographic characteristics, comorbidities, and clinical outcomes are summarized in Table 4.

**Table 4:** Distribution of demographic characteristics, comorbidities, and clinical outcomes according to serum potassium levels

Variables (*N* = 998)	Hypokalemia^1^ (*n* = 93)	Normokalemia^2^ (*n *= 796)	Hyperkalemia^3^ (*n* = 109)	*p*-value	Post-hoc*
Age, years	78 ± 13	71 ± 16	73 ± 14	< 0.001	1–2; 1–3
Sex, n (%)				0.330	
Female	45 (48.4)	371 (46.6)	43 (39.4)		
Male	48 (51.6)	425 (53.4)	66 (60.6)		
ACE inhibitor use, n (%)	10 (10.8)	145 (18.2)	20 (18.3)	0.196	
Diuretic use, n (%)	15 (16.1)	128 (16.1)	35 (32.1)	< 0.001	
Chronic lung disease, n (%)	46 (49.5)	391 (49.1)	57 (52.3)	0.824	
Diabetes mellitus, n (%)	21 (22.6)	224 (28.2)	33 (30.3)	0.439	
Chronic heart failure, n (%)	30 (32.3)	148 (18.6)	39 (35.8)	< 0.001	
Chronic kidney disease, n (%)	9 (9.7)	58 (7.3)	19 (17.4)	0.002	
History of malignancy, n (%)	8 (8.6)	41 (5.2)	6 (5.5)	0.386	
Immunosuppression, n (%)	8 (8.6)	53 (6.7)	9 (8.3)	0.680	
Aspiration pneumonia, n (%)	17 (18.3)	74 (9.3)	2 (1.8)	< 0.001	
Nursing home residence, n (%)	2 (2.2)	16 (2.0)	1 (0.9)	0.724	
Functional Status, n (%)	25 (26.9)	128 (16.1)	6 (5.5)	< 0.001	
CURB-65 score, n (%)				< 0.001	
0–1	16 (17.2)	333 (41.8)	32 (29.4)		
2	41 (44.1)	303 (38.1)	47 (43.1)		
≥ 3	36 (38.7)	160 (20.1)	30 (27.5)		
History of pneumonia, n (%)	25 (26.9)	201 (25.3)	35 (32.1)	0.307	
Macrolide use, n (%)	28 (30.1)	396 (49.7)	54 (49.5)	0.001	
Days of macrolide therapy	5.9 ± 2.6	6.2 ± 3.1	6.9 ± 2.7	0.205	
Days of empirical antibiotic therapy	6 (4–8)	6 (4–8)	7 (5–9)	0.105	
Carbapenem resistance, n (%)	5 (15.2)	26 (8.9)	4 (8.7)	0.500	
ICU admission, n (%)	27 (29.0)	90 (11.3)	27 (24.8)	< 0.001	
Need for invasive mechanical ventilation, n (%)	16 (17.2)	36 (4.5)	16 (14.7)	< 0.001	
Need for non-invasive ventilation, n (%)	7 (7.5)	70 (8.8)	16 (14.7)	0.115	
Length of hospital stay, days	6 (4–10)	6 (4–9)	7 (4–10)	0.353	
In-hospital mortality, n (%)	14 (15.1)	33 (4.1)	15 (13.8)	< 0.001	
6-month mortality, n (%)	7 (7.5)	41 (5.2)	15 (13.8)	0.002	

Continuous variables are presented as mean ± standard deviation or median (interquartile range), as appropriate. Comparisons among the three groups were performed using one-way ANOVA with Bonferroni correction, Welch ANOVA with Games–Howell post-hoc test, or Kruskal–Wallis test with Dunn–Bonferroni correction, depending on data distribution and variance homogeneity. Categorical variables were compared using the chi-square test; Fisher’s exact test or Monte Carlo exact test was applied when expected cell counts were insufficient.

Age differed significantly among potassium groups (*p* < 0.001), with post-hoc analyses showing that patients with hypokalemia were significantly older than both normokalemic and hyperkalemic patients. Diuretic use varied across groups (*p* < 0.001) and was most frequent in the hyperkalemia group. The prevalence of chronic heart failure differed significantly (*p* < 0.001), being higher in both hypokalemia and hyperkalemia groups compared with normokalemia. Chronic kidney disease was significantly more frequent among patients with hyperkalemia (*p* = 0.002).

Markers of disease severity also differed among groups. CURB-65 score distribution varied significantly (*p* < 0.001), with higher scores observed more frequently in patients with potassium abnormalities. Rates of intensive care unit admission differed significantly (*p* < 0.001) and were higher in patients with hypokalemia and hyperkalemia. Similarly, invasive mechanical ventilation was more frequently required in both hypokalemic and hyperkalemic patients compared with normokalemic patients (*p* < 0.001).

In-hospital mortality differed significantly among groups (*p* < 0.001), with higher mortality rates observed in patients with hypokalemia (15.1%) and hyperkalemia (13.8%) compared with normokalemia (4.1%). Six-month mortality was also significantly higher in patients with potassium abnormalities (*p* = 0.002).

### Laboratory findings

Admission laboratory parameters according to potassium categories are presented in Table 5. Estimated glomerular filtration rate differed significantly among groups (*p* = 0.005), with lower values observed in patients with hyperkalemia. Serum creatinine levels also differed significantly (*p* = 0.002), with higher values in the hyperkalemia group compared with both hypokalemia and normokalemia groups.

**Table 5:** Distribution of admission laboratory parameters according to serum potassium levels

Variables (*N* = 998)	Hypokalemia^1^ (*n* = 93)	Normokalemia^2^ (*n* = 796)	Hyperkalemia^3^ (*n* = 109)	*p*-value	Post-hoc*
Estimated glomerular filtration rate (eGFR), mL/min/1.73 m^2^	62.2 ± 27.2	69.0 ± 30.7	60.5 ± 29.3	0.005	2–3
Serum creatinine, mg/dL	1.0 (0.8–1.5)	1.0 (0.8–1.2)	1.1 (0.9–1.5)	0.002	1–3; 2–3
Serum albumin, g/L	31 (26–34)	29 (25–34)	30 (25–34)	0.492	
Serum osmolality, mOsm/kg	256.5 (244–262)	267 (259–275.2)	266 (259–277.8)	0.173	
Serum sodium, mmol/L	141 (139–144)	139 (136–142)	137 (134–141)	< 0.001	1–2; 1–3; 2–3
C-reactive protein (CRP), mg/L	109.3 ± 73.4	101.2 ± 78.5	100.0 ± 81.4	0.626	
Procalcitonin, ng/mL	0.7 (0.1–2.1)	0.1 (0–0.7)	0.6 (0.1–1.3)	< 0.001	1–2; 2–3
Arterial pH	7.4 (7.3–7.5)	7.4 (7.4–7.5)	7.4 (7.3–7.4)	0.019	1–3; 2–3
Serum lactate, mmol/L	1.6 (1.1–2.8)	1.5 (1.1–2.3)	1.7 (1.2–2.3)	0.529	

Continuous variables are presented as mean ± standard deviation or median (interquartile range), as appropriate. Comparisons among the three groups were performed using one-way ANOVA with Bonferroni correction, Welch ANOVA with Games–Howell post-hoc test, or Kruskal–Wallis test with Dunn–Bonferroni correction, depending on data distribution and variance homogeneity

*Abbreviations*: *eGFR* estimated glomerular filtration rate, *CRP* C-reactive protein

Serum sodium levels varied significantly across potassium groups (*p* < 0.001), with lower sodium levels observed in patients with hyperkalemia. Procalcitonin levels differed significantly (*p* < 0.001) and were higher in both hypokalemic and hyperkalemic patients compared with normokalemic patients. Arterial pH also differed significantly among groups (*p* = 0.019), whereas no significant differences were observed in C-reactive protein or lactate levels (all *p* > 0.05).

Multivariable logistic regression analyses were performed to identify independent factors associated with electrolyte disturbances at hospital admission (Tables 6 and 7).

**Table 6:** Independent risk factors associated with dysnatremia at hospital admission

Variables	Hyponatremia OR (95% CI)	*p*-value	Hypernatremia OR (95% CI)	*p*-value
Age, years	1.018 (0.997–1.039)	0.087	1.012 (0.984–1.042)	0.401
Sex				
Female	Reference	–	Reference	–
Male	1.130 (0.727–1.756)	0.587	0.606 (0.334–1.098)	0.099
CURB-65 score				
0–1	Reference	–	Reference	–
2	0.697 (0.417–1.164)	0.168	1.444 (0.671–3.107)	0.348
≥ 3	1.405 (0.812–2.431)	0.225	2.166 (0.951–4.931)	0.066
Diuretic use	**3.089 (1.879–5.080)**	** < 0.001**	**2.577 (1.307–5.079)**	**0.006**
Chronic heart failure	1.205 (0.716–2.026)	0.483	1.117 (0.562–2.220)	0.751
Serum albumin, g/L	0.998 (0.987–1.010)	0.779	1.001 (0.989–1.013)	0.847
eGFR, mL/min/1.73 m^2^	1.009 (0.999–1.020)	0.086	0.997 (0.983–1.010)	0.635
Diabetes mellitus	0.896 (0.555–1.448)	0.655	**0.488 (0.239–0.998)**	**0.049**

Multivariable logistic regression analyses were performed to identify independent factors associated with hyponatremia and hypernatremia at hospital admission. Results are presented as odds ratios (ORs) with 95% confidence intervals (CIs)

*Abbreviations*: *OR* Odds ratio, *CI* Confidence interval, *eGFR* estimated glomerular filtration rate

**Table 7:** Independent risk factors associated with dyskalemia at hospital admission

Variables	Hypokalemia OR (95% CI)	*p*-value	Hyperkalemia OR (95% CI)	*p*-value
Age, years	**1.043 (1.012–1.076)**	**0.007**	**0.969 (0.947–0.992)**	**0.008**
Sex				
Female	Reference	–	Reference	–
Male	1.425 (0.794–2.555)	0.235	**1.973 (1.140–3.415)**	**0.015**
CURB-65 score				
0–1	Reference	–	Reference	–
2	1.858 (0.860–4.010)	0.115	1.235 (0.655–2.331)	0.514
≥ 3	**2.968 (1.327–6.640)**	**0.008**	1.986 (0.990–3.984)	0.053
ACE inhibitor use	—	—	0.851 (0.426–1.701)	0.647
Diuretic use	0.674 (0.297–1.529)	0.345	**2.731 (1.454–5.131)**	**0.002**
Chronic heart failure	**2.079 (1.108–3.901)**	**0.023**	**2.089 (1.169–3.731)**	**0.013**
Serum albumin, g/L	1.026 (0.981–1.073)	0.256	1.019 (0.981–1.057)	0.329
eGFR, mL/min/1.73 m^2^	1.005 (0.991–1.019)	0.482	**0.984 (0.973–0.995)**	**0.006**

Multivariable logistic regression analyses were performed to identify independent factors associated with hypokalemia and hyperkalemia at hospital admission. Results are presented as odds ratios (ORs) with 95% confidence intervals (CIs)

*Abbreviations*: *OR* Odds ratio, *CI* Confidence interval, *eGFR* estimated glomerular filtration rate

For hyponatremia, diuretic use was the only variable independently associated with its presence (OR 3.089, 95% CI 1.879–5.080; *p* < 0.001). (Table 6).

Regarding hypernatremia, diuretic use was independently associated with an increased likelihood of hypernatremia (OR 2.577, 95% CI 1.307–5.079; *p* = 0.006), whereas diabetes mellitus was inversely associated (OR 0.488, 95% CI 0.239–0.998; *p* = 0.049). (Table 6).

In the analysis of hypokalemia, increasing age (OR 1.043, 95% CI 1.012–1.076; *p* = 0.007), a CURB-65 score ≥ 3 (OR 2.968, 95% CI 1.327–6.640; *p* = 0.008), and the presence of chronic heart failure (OR 2.079, 95% CI 1.108–3.901; *p* = 0.023) were independently associated with hypokalemia. (Table 7).

For hyperkalemia, male sex (OR 1.973, 95% CI 1.140–3.415; *p* = 0.015), diuretic use (OR 2.731, 95% CI 1.454–5.131; *p* = 0.002), chronic heart failure (OR 2.089, 95% CI 1.169–3.731; *p* = 0.013), and lower estimated glomerular filtration rate (OR 0.984, 95% CI 0.973–0.995; *p* = 0.006) were independently associated with hyperkalemia. Increasing age was inversely associated with hyperkalemia (OR 0.969, 95% CI 0.947–0.992; *p* = 0.008) (Table 7).

### Microbiological findings

Sputum cultures were obtained in 334 patients (33.5% of the study population). No bacterial growth was detected in 225 cases (67.4%). Among patients with positive sputum cultures, the most frequently isolated pathogens were *Pseudomonas spp.* (*n* = 21), *Acinetobacter spp.* (*n* = 12), *Klebsiella pneumoniae* (*n* = 12), *Stenotrophomonas spp.* (*n* = 7), and *Escherichia coli* (*n* = 6). Less frequently isolated organisms included *Staphylococcus aureus* (*n* = 2), *Enterobacter spp.* (*n* = 3), streptococcal species (*n* = 9), and *Candida* species (*n* = 13). Polymicrobial growth was observed in eight patients, while ten cultures were considered contaminated.

Blood cultures were obtained in 141 patients (14.1%), of whom seven (5.0%) yielded positive results. Methicillin-resistant *Staphylococcus aureus* (MRSA) was isolated in two patients, Gram-negative bacilli in three patients, and Gram-positive cocci in two patients.

## Discussion

In this retrospective cohort of 998 adults hospitalized with community-acquired pneumonia (CAP), electrolyte disturbances at admission were common. Hyponatremia was the most frequent abnormality, whereas hypernatremia or potassium disorders were less frequent but clustered with markers of higher clinical severity. Overall, hypernatremia was associated with increased ICU admission, invasive mechanical ventilation requirement, longer hospital stay, and higher in-hospital mortality, while hypokalemia and hyperkalemia were associated with higher ICU use and mortality compared with normal potassium levels. These findings suggest that admission dysnatremia and dyskalemia may reflect a more vulnerable host phenotype or more severe acute illness in hospitalized CAP. Electrolyte disturbances were managed according to standard clinical practice and established principles; however, specific treatment strategies and their impact on outcomes were not systematically analyzed in this study [9].

### Sodium disturbances in CAP

Hyponatremia is frequently observed in CAP and is commonly attributed to inappropriate antidiuresis (SIAD), hypovolemia, or other non-osmotic stimuli for vasopressin secretion during infection. Aetiologic work in CAP has shown that SIAD and hypovolemic hyponatremia may account for a substantial proportion of cases [10]. In our cohort, hyponatremia was common and was associated with a higher prevalence of chronic heart failure and more frequent diuretic exposure, suggesting that comorbidity burden and medication use may contribute to low sodium at presentation. However, hyponatremia was not associated with higher in-hospital or 6-month mortality, and it showed no significant association with ICU admission or invasive ventilation in our dataset. This is consistent with the concept that, in some CAP populations, hyponatremia may act as a marker of underlying comorbidity or volume status rather than a direct marker of pneumonia severity.

In contrast, hypernatremia although less frequent showed the strongest association with adverse short term outcomes in our study, including higher ICU admission, invasive ventilation, and in-hospital mortality. Hypernatremia in acute illness often reflects dehydration, reduced oral intake, impaired thirst, or altered mental status and dependence, which may be particularly relevant in older or bed-dependent patients. Our findings align with recent CAP-focused data indicating that hypernatremia is linked to worse outcomes and may outperform hyponatremia in predicting mortality in hospitalized CAP [11]. Importantly, these associations should be interpreted as reflective of illness severity and host vulnerability rather than as causal effects of sodium disturbances.

### Potassium disturbances and clinical severity

Potassium abnormalities were also associated with clinically relevant outcomes. Both hypokalemia and hyperkalemia were linked to greater ICU utilization and higher in-hospital and 6-month mortality compared with normokalemia. Hyperkalemia clustered with chronic kidney disease and chronic heart failure, along with laboratory features compatible with more profound systemic stress (higher procalcitonin and lower arterial pH), suggesting that renal dysfunction and metabolic derangements may contribute to potassium elevation during CAP [9, 12, 13]. Our observations are consistent with broader cohort evidence demonstrating that hyperkalemia is associated with increased mortality, cardiovascular events, hospitalizations, and ICU admissions. This pattern is biologically expected, as metabolic acidosis promotes extracellular potassium shift, and therefore these findings likely reflect underlying physiological stress rather than an independent causal effect.

Hypokalemia, on the other hand, was associated with older age, higher CURB-65 scores, and chronic heart failure in multivariable analyses. In critically ill populations, potassium disturbances are clinically important because they may contribute to arrhythmias, respiratory muscle weakness, and difficulty in ventilator liberation, and thus may coincide with higher levels of supportive care [10]. Although our retrospective dataset cannot establish causality, the consistent association of potassium abnormalities with ICU admission and invasive ventilation supports the interpretation that potassium derangements at admission may accompany a more severe physiological presentation.

### Independent factors associated with electrolyte abnormalities

In multivariable models, diuretic use was independently associated with both hyponatremia and hypernatremia, underscoring the importance of medication exposure and fluid balance in shaping sodium levels at hospital presentation. For potassium disorders, chronic heart failure, renal function (eGFR), sex, and illness severity (CURB-65) emerged as relevant factors, which is biologically plausible given the interplay between neurohormonal activation, renal potassium handling, and acute systemic stress in pneumonia and critical illness [10]. An important limitation related to disease severity assessment is the use of the CURB-65 score. Although CURB-65 is widely used in clinical practice, it lacks granularity, and some of its components may be influenced by chronic comorbid conditions rather than acute infection severity. In particular, elevated urea levels may reflect underlying chronic renal dysfunction in addition to acute illness, which may affect the interpretation of the score in hospitalized patients. Electrolyte disturbance categories in this study were defined based on admission values and were not mutually exclusive; therefore, a subset of patients presented with concurrent sodium and potassium abnormalities, which may have contributed to worse clinical outcomes.

### Macrolide use in our cohort

Approximately half of the patients in our cohort received macrolide therapy. While this proportion might initially seem high if interpreted solely as an indicator of atypical pathogen prevalence, it should be considered within the context of contemporary treatment strategies for hospitalized community-acquired pneumonia. Current clinical guidelines recommend macrolides as part of empiric combination therapy, particularly in hospitalized patients, severe CAP, or situations where broader antimicrobial coverage is required [6, 14]. In addition to their antimicrobial activity against atypical pathogens, macrolides have been shown to exert immunomodulatory effects, which may contribute to improved clinical outcomes. Previous studies and meta-analyses have reported favorable outcomes associated with β-lactam–macrolide combination therapy compared with β-lactam monotherapy in selected hospitalized CAP populations. Therefore, the frequency of macrolide use observed in this study is more likely to reflect guideline-consistent empiric treatment practices rather than a high confirmed prevalence of atypical pneumonia.

### Limitations

This study benefits from a relatively large hospitalized CAP cohort and systematic assessment of both sodium and potassium disturbances at admission, with clinically relevant outcomes including ICU admission, ventilatory support, in-hospital mortality, and 6-month mortality. Nonetheless, limitations include the retrospective single-center design, potential residual confounding (e.g., hydration status, diuretic dose, nutritional status), and the lack of serial electrolyte measurements to evaluate persistence, correction, or in-hospital trajectories. In addition, due to the retrospective design, detailed information regarding indications for respiratory support modalities or treatment limitation could not be consistently assessed. Selection bias may also exist because cultures and advanced testing may have been performed more often in severe cases. Finally, because some patients may have had concurrent electrolyte abnormalities, overlapping exposure could contribute to observed associations; future analyses could explicitly quantify and model combined electrolyte derangements.

### Clinical implications

From a clinical perspective, admission sodium and potassium abnormalities in hospitalized CAP should be viewed as readily available and low-cost markers of physiological vulnerability. Rather than independent therapeutic targets, these disturbances provide contextual information about acute illness burden, hydration status, renal function, and comorbidity load.

Routine assessment of electrolyte abnormalities may therefore complement established severity scores and support early risk stratification. In older patients and those requiring higher levels of supportive care, recognition of dysnatremia or dyskalemia may prompt closer monitoring and timely correction of reversible contributing factors.

## Conclusion

In this large retrospective cohort of hospitalized patients with community-acquired pneumonia, admission sodium and potassium abnormalities were common and prognostically informative. Hypernatremia and potassium disturbances were consistently associated with greater illness severity, higher rates of intensive care use and mechanical ventilation, and increased short- and medium-term mortality. In contrast, hyponatremia appeared to reflect comorbidity burden and treatment-related factors rather than pneumonia severity itself.

Overall, admission electrolyte abnormalities in CAP should be interpreted primarily as readily available markers of physiological stress and host vulnerability rather than independent therapeutic targets. Their early identification may complement established severity assessments and support timely risk stratification and clinical vigilance during hospitalization.

## Supplementary Information

Additional file 1.

Additional file 2.

## Data Availability

The datasets generated and/or analyzed during the current study are available from the corresponding author on reasonable request.

## Abbreviations

ACE: Angiotensin-converting enzyme
COVID-19: Coronavirus disease 2019
CURB-65: Confusion, Urea, Respiratory rate, Blood pressure, and age ≥ 65 years
CAP: Community-acquired pneumonia
ICU: Intensive care unit
MV: Mechanical ventilation
SIADH: Syndrome of inappropriate antidiuretic hormone secretion
ATS: American Thoracic Society
IDSA: Infectious Diseases Society of America
CRP: C-reactive protein
eGFR: Estimated glomerular filtration rate
ANOVA: Analysis of variance
IQR: Interquartile range
OR: Odds ratio
CI: Confidence interval
SPSS: Statistical Package for the Social Sciences
MRSA: Methicillin-resistant Staphylococcus aureus
SIAD: Syndrome of inappropriate antidiuresis
