# Supplementary material for: Risk factors and clinical impact of sodium and potassium disorders in community-acquired pneumonia
Source: BMC Pulm Med. 2026 Apr 29;26:270. doi: 10.1186/s12890-026-04322-y (PMC13270646; doi:10.1186/s12890-026-04322-y)
Supplement: Supplementary file 1 — Additional file 1. [file 12890_2026_4322_MOESM1_ESM.docx]

# STROBE Checklist

**Manuscript Title:** Risk Factors and Clinical Impact of Sodium and Potassium Disorders in Community-Acquired Pneumonia

| Item No | STROBE Recommendation | Page Number |
| --- | --- | --- |
| 1 | Indicate study design in title/abstract | 1. |
| 2 | Explain scientific background and rationale | 2. |
| 3 | State specific objectives | (Page 2) Introduction – last paragraph |
| 4 | Present key elements of study design | (Page 2-3) Methods – first paragraph |
| 5 | Describe setting and dates | (Page 2) Methods – first paragraph |
| 6 | Participants eligibility criteria and selection | (Page 4) Methods |
| 7 | Clearly define outcomes and exposures | (Page 5) Methods |
| 8 | Data sources and measurement methods | (Page 3-4) Methods |
| 9 | Describe efforts to address potential bias | (Page 5-6) Methods  (Page 20) Limitations |
| 10 | Explain how study size was determined | (Page 4) Methods |
| 11 | Explain handling of quantitative variables | (Page 5-6) Methods – Statistical Analysis |
| 12 | Describe all statistical methods | (Page 5-6) Methods – Statistical Analysis |
| 13 | Report participant flow (e.g., flow diagram) | (Page 4) Methods |
| 14 | Provide descriptive data | (Page 5-6)Results – Baseline Characteristics  Tables 1–4 |
| 15 | Report outcome data | (Page 15-17)Results – Tables 5,6–7 |
| 16 | Present main results with estimates | (Page 15-17)Results – Tables 5,6–7 |
| 17 | Report other analyses (e.g., subgroups) | (Page 15-17)Results – Tables 5,6–7 |
| 18 | Summarize key results | (Page 17) Discussion – first paragraph |
| 19 | Discuss study limitations | (Page 17)Discussion – limitations |
| 20 | Provide cautious interpretation | (Page 20) Discussion – conclusion |
| 21 | Discuss generalisability | (Page 20)Discussion – conclusion |
| 22 | Describe funding source | (Page 21)Declarations – Funding section |
