# Supplementary material for: Risk factors and clinical impact of sodium and potassium disorders in community-acquired pneumonia
Source: BMC Pulm Med. 2026 Apr 29;26:270. doi: 10.1186/s12890-026-04322-y (PMC13270646; doi:10.1186/s12890-026-04322-y)
Supplement: Supplementary file 2 — Additional file 2. [file 12890_2026_4322_MOESM2_ESM.docx]

**Supplementary Table 1.**Multicollinearity diagnostics for multivariable logistic regression models

| **Model** | **Max VIF** | **Min Tolerance** |
| --- | --- | --- |
| **Table 5** | 2,238 | 0,447 |
| **Table 6** | 2,179 | 0,461 |
| **Table 7** | 2,184 | 0,458 |
| **Table 8** | 2,176 | 0,460 |

All maximum VIF values were below 2.5 and all tolerance values were above 0.40, indicating no evidence of clinically relevant multicollinearity.
